# Maternal and Child Health Pipeline Training Programs: A Description of Training Across 6 Funded Programs

**DOI:** 10.1007/s10995-022-03375-9

**Published:** 2022-03-14

**Authors:** V. Moerchen, L. Taylor-DeOliveira, M. Dietrich, A. Armstrong, J. Azeredo, H. Belcher, N. Copeland-Linder, P. Fernandes, A. Kuo, C. Noble, O. Olaleye, H. Salihu, C. R. Waters, C. Brown, M. M. Reddy

**Affiliations:** 1 University of Wisconsin-Milwaukee, 3409 N Downer Ave, Pavilion 366, Milwaukee, WI 53211 USA; 2 University of South Florida, Tampa, FL USA; 3 Kennedy Krieger Institute, Johns Hopkins University, Baltimore, MD USA; 4 University of Southern California, Los Angeles, CA USA; 5 University of North Texas, Fort Worth, TX USA; 6 Texas Southern University, Houston, TX USA; 7 Baylor College of Medicine, Houston, TX USA; 8 Alabama State University, Montgomery, AL USA; 9 (MR)U.S. Department of Health and Human Services, Health Resources and Services Administration, Maternal and Child Health Bureau, Rockville, MD USA

**Keywords:** Maternal and child health training programs, Pipeline, Under-represented, Workforce development, Undergraduate

## Abstract

**Purpose:**

The HRSA-funded maternal and child health pipeline training programs (MCHPTPs) are a response to the critical need to diversify the MCH workforce, as a strategy to reduce health disparities in MCH populations. These MCHPTPs support students from undergraduate to graduate education and ultimately into the MCH workforce.

**Description:**

The models and components of training across the six MCHPTPs funded in 2016–2021 are summarized, to examine the design and delivery of undergraduate pipeline training and the insights gained across programs.

**Assessment:**

Strategies that emerged across training programs were organized into three themes: recruitment, support for student persistence (in education), and pipeline-to-workforce intentionality. Support for student persistence included financial support, mentoring, creating opportunity for students to develop a sense of belonging, and the use of research as a tool to promote learning and competitiveness for graduate education. Finally, the link to Maternal and Child Health Bureau (MCHB) long-term training and other MCHB opportunities for professional development contributed significant nuance to the pipeline-to-workforce objectives of these programs.

**Conclusions:**

The MCHPTPs not only increase the diversity of the MCH workforce, they also actively prepare the next generation of MCH leaders. The intentional connection of undergraduates to the infrastructure and continuum of MCH training, underscores the comprehensive impact of this funding.

## Significance

Increasing the diversity of the MCH workforce is a priority in the national effort to eliminate health disparities. MCHB initiated pipeline training programs in 2006 to attract and support undergraduate students from underrepresented and disadvantaged backgrounds to pursue careers in MCH public health and MCH-related health professions. We provide a comprehensive summary of the six funded HRSA-funded MCH pipeline training programs (MCHPTPs, 2016–2021) to examine the design and delivery of these programs that collectively function to support the pipeline-to-workforce focus of this funding.

## Purpose

The need for a diverse and representative healthcare workforce has never been more urgent. The gross under-representation of racial and ethnic minorities in healthcare is increasing, as the demographic of the United States evolves toward a majority-minority population (Colby & Ortman, 2014). Achieving a healthcare workforce that is racially- and culturally-concordant with the community it serves is critical; without it, health disparities may be exacerbated. Increasing the diversity of the healthcare workforce is one strategy to eliminate health disparities by reducing cultural and linguistic barriers, increasing access and utilization of healthcare, and enhancing the healthcare experience (LaVeist & Pierre, 2014; Mitchell & Lassister, 2006). Pipeline programs, in general, are intended to increase the number of individuals from under-represented minorities (URMs) in healthcare by increasing awareness of healthcare fields and reducing gaps in educational attainment (Bouye et al., 2016; Katz et al., 2016; Smith et al., 2009a, 2009b). Given the ongoing, fast-paced demographic shifts of the United States’ population and the growing impact of health disparities on our minority populations, pipeline programs are critical to social justice in health care and the current and future population health of the country (Bouye et al., 2016).

Historically, two important publications called for a focus on the educational pipeline to healthcare careers for URM students, as a critical strategy to address workforce disparities. *Missing Persons: Minorities in the Health Professions* (The Sullivan Commission, 2004) and *In the Nation’s Compelling Interest: Ensuring Diversity in the Healthcare Workforce* (Institute of Medicine, 2004), addressed under-representation in healthcare and provided recommendations for diversity training programs to increase URM student access to healthcare education. These recommendations included innovative programs to support URM student intention for healthcare careers and persistence along the academic pathways to these careers. Academic enrichment programs and services including mentoring, test-taking skill development, and guidance with components and processes for graduate/medical school application were identified as baseline components of these programs.

In 2006, the Health Resources and Services Administration (HRSA) initiated funding for undergraduate MCH pipeline training that continues today. This funded initiative was called MCH Pipeline from 2006 to 2021 and is now referred to as Leadership Education and Advancement in Undergraduate Pathways (LEAP) for funding from 2021 forward. These Pipeline/LEAP programs specifically address the critical lack of diversity in the MCH workforce with the goal of ensuring the health of mothers, children and families from underserved and vulnerable populations. The purpose of the HRSA-funded MCH Pipeline Training Program (MCHPTP) is “to promote the development of a culturally diverse and representative health care workforce by recruiting undergraduate students from economically and educationally disadvantaged and racially/ethnically diverse backgrounds into maternal and child health (MCH) professions” (MCHB/NOFO, 2016, p 1). Economic and educational disadvantage include growing up in an educationally underserved community, having received services and supports due to low socioeconomic status, and having the potential to be first generation college educated. Racial and ethnic diversity for this program is defined as African American, Hispanic/Latino, Asian, Hawaiian/Pacific Islander, and American Indian/Alaskan Native.

From 2007 to 2019, 5053 undergraduate students completed MCH Pipeline Training across a total of 7 funded programs. In the 2016–2021 funding cycle, there were six MCHPTPs located in Alabama, California, Florida, Maryland, Texas, and Wisconsin. All programs were housed at universities that serve a large proportion of or are committed to supporting equitable educational opportunities for students from historically under-represented groups. In 2019, 67% of the trainees were racially diverse plus 19.5% were Hispanic/Latino (HRSA, MCHB, DGIS, 2020). Fourteen percent of faculty and staff were from underrepresented groups (HRSA, MCHB, DGIS, 2020).

The MCHPTPs are extracurricular traineeships in which undergraduate students participate outside their major/degree area(s). Students admitted as trainees to an MCHPTP demonstrate interest in and intention to pursue a career in MCH public health and related fields such as pediatric medicine or dentistry, MCH nutrition, MCH social work, MCH nursing, psychology, health education, pediatric occupational therapy, pediatric physical therapy, and speech language pathology. Research has shown that healthcare career intentions among URM students may not survive undergraduate education (Hurtado et al., 2008). The MCHPTP responds to this threat by supporting the persistence of career and educational intention among URM students. MCHPTPs, therefore, include provision of enriching experiences, academic support, mentorship, education in MCH public health, research exposure, leadership opportunities, clinical and/or field experiences, and preparation for graduate school application and progression to the MCH workforce. By supporting academic retention and mentoring trainees for graduate school readiness, the MCHPTP functions to expand access to MCH healthcare and MCH public health fields for URM students. Remarkably, 76% of MCHPTP “program completers” have gone on to enter graduate programs and work with MCH populations (HRSA, MCHB, DGIS, 2020), achieving the pipeline-to-workforce intention of this training.

The purpose of this paper is to describe and discuss the models and training components of the MCHPTPs funded 2016–2021. The comprehensiveness of these programs is highlighted and explored, and aspects of training unique or nuanced to MCH are also identified. Understanding the design and delivery of pipeline training programs to support educational attainment and workforce outcomes requires a deeper review of the intentionality of the structure of these programs. This paper begins such a review.

## Description

This report provides a descriptive overview of the structure of the six MCHPTPs funded during 2016–2021. Three of the MCHPTPs had been funded since the 2006 inception of this MCHB initiative, and three were newly funded from 2016 to 2021. This paper was a collaborative effort between the program directors and faculty of the six programs and the MCHB project officers. While two of the MCHPTPs from the first cycle of funding have been previously described (Guerroro et al., 2015; Kuo et al., 2015; Pizur-Barnekow et al., 2010), the absence of a composite summary across programs represented a significant gap in the literature on this HRSA/MCHB initiative. This paper addresses this gap by providing a detailed description of the six MCHPTPs.

The design of each MCHPTP is guided broadly by the goals of the Division of Maternal and Child Health Workforce Development: (1) to achieve MCH workforce and leadership development, (2) to prepare and empower MCH leaders to promote health equity, (3) to foster interdisciplinary/interprofessional development of tomorrow’s MCH workforce, and (4) to contribute to science, innovation and quality improvement (MCHB/NFO, 2016). To support these goals, the MCHPTPs are required to provide a training curriculum that (a) includes appropriate undergraduate didactic, research, clinical and/or field experiences, (b) exposes students to the breadth of MCH professions and state Title V agencies, (c) promotes and engages awareness of emerging MCH health problems and practice issues, and (d) incorporates linkages to long-term MCH graduate training programs. Each program is then designed based on the unique strengths of each program’s campus and the needs of the communities they serve, while still meeting the funding requirements specified in the notice of funding opportunity (MCHB/NOFO, 2016). Capturing this variation across programs or the different means to similar ends of this training is critical to understanding how programs like the MCHPTPs achieve their impact.

The pipeline (now LEAP) initiative is still relatively new in the history of MCHB training programs. However, the design of MCHPTPs is reflective of the long history of graduate-level MCH training (Athey et al., 2000). The benefit of this history is that in addition to *pipeline* elements of mentoring, enrichment, and academic support, MCHPTP undergraduate students are also exposed to leadership development, interdisciplinary training and practice, cultural/linguistic competence, and emerging issues in MCH public health (MCHB/NOFO, 2016). Each MCHPTP must also be linked to at least one long-term MCH graduate training program, so that the continuum of MCH training is apparent to the undergraduate students in the pipeline programs.

MCHB resources also contribute to the structure of the MCHPTPs. The availability of the MCH Leadership Competencies (U.S. Department of Health and Human Services, 2018) provides a critical “roadmap of sorts” for career-long professional development that may be unique to MCH training, compared to other pipeline programs. Other aspects of MCHB supports for training include the Making Lifelong Connections conference that assists emerging MCH leaders with developing their own network of MCH colleagues, and the opportunity for undergraduate Pipeline trainees to be paired with graduate trainees from MCH Centers of Excellence through the Title V Summer Internship (Handler et al., 2018).

The above guidelines, requirements, and opportunities in addition to the goals and directives of the funding NOFO helped shape the categories of program descriptors determined by consensus across program directors to achieve a descriptive summary of these MCHPTPs. An internal survey of programs was then administered electronically to program directors to capture the details of training at each site and the response of the programs to MCH specific requirements and opportunities, as well as reflections from experience administering the MCHPTPs. Once the survey results were summarized, each program had the opportunity to respond to details for their program to assure completeness and accuracy of reporting.

## Assessment

Table 1 provides a broad description of each MCHPTP, encompassing the length/duration of the program, the educational levels of students in the program, the size of the program, and the academic majors and intended careers of students in the program. Additionally, recruitment strategies are captured to address a key element in sustaining pipeline training.

**Table 1:** Models of undergraduate HRSA-funded MCH pipeline training programs

Program	Duration	Student level	Model	#Students/cohort or/year	Undergraduate majors	Target careers	How recruited
ASU	1 year +	Fr	Cohort	25	Students with interests in careers with women and children	Public health, medical rehab, psychology, social work, education	Campus/community outreach; class visits, social media, student referrals
BCM—TSU	1 year	Fr–Jr	Cohort	20	Health admin, pre-pharmacy, accounting, biological sciences, psychology, pre-medicine, physics, engineering, health information management health education, chemistry, education	Pharmacy, medicine, healthcare, public health	Direct recruitment^a^; class visits; student referrals; emails to advisors
KKI	1 year	Fr–Jr	Cohort	10	Public health, social sciences	Public health, medicine, psychology	Center for diversity website; outreach to local HBCUs and other colleges
UCLA	4 years	Fr–Sr	Other	15–20	Biological sciences, physiological sciences	Medicine, public health, nursing, physician assistant	Campus organizations for diversity; outreach to student and community organizations
USF	2 years	So–Jr	Cohort	12	Biomedical sciences, biomedical engineering, health sciences, psychology, pre-nursing, public health	Health administration, epidemiology, medicine nursing, law, child psychology	Class visits; direct recruitment^a^; flyers; presentations w feeder programs; outreach to student organizations
UWM	4 + years	Fr–Sr	Cohort (Jr/Sr) + Other (Fr/So)	26 +	Communication sciences/disorders, kinesiology /pre-physical therapy, nursing, nutrition, occupational sciences and tech/pre-occupational therapy, pre-medicine, pre-public health, psychology, social work	Medicine, nursing, nutrition/dietetics, occupational therapy, physical therapy, psychology, public health, social work, speech-language pathology﻿	Campus office of diversity and inclusion; class visits; direct recruitment^a^; electronic banners; flyers; outreach to student organizations; student referral; university advising

*ASU* Alabama State University, *BCM-TSU* Baylor College of Medicine and Texas Southern University-Houston, *KKI* Kennedy-Krieger Institute, *UCLA* University of California-Los Angeles, *USF* University of South Florida, *UWM* University of Wisconsin-Milwaukee, *Fr* Freshman, *So* Sophomore, *Jr* Junior, *Sr* Senior, *Cohort* a model where a group of students goes through training together, *Other* a model where students are part of the larger program, but not necessarily progressing through the program as part of a defined group

^a^Direct recruitment occurs with 1:1 contact between a program faculty or staff member and a prospective student

Table 2 captures the themes that emerged from the summary of the MCHPTP programs related to components of training that support student retention or persistence while also achieving the MCH public health exposure that is significant to the MCH workforce and professional development.

**Table 2:** Components of training to support retention and pipeline-to-workforce

Program	Academic supports	Required courses/training	Community engagement	Research	Conference presentation (s)	Support of professional development
ASU	Advisors mentors	Summer institute	Community health resources fair; service learning; community outreach	No	N/A	Test prep
BCM-TSU	Academic advising; research mentors	Integrated MCH foundation and research course	Community outreach opportunities; health fairs	Research course that leads to a manuscript/publication	University research week (annual); professional conferences	Professional organization (Global Alliance for MCH) conference attendance
KKI	Research mentor	Four 10-week seminars: 1. Research 2. Professional development 3. CDC winnable battles 4. Prevention of health disparities across the life course	“Families as Faculty” program	10-week research project	University research conferences; national conferences	Dual culturally congruent mentoring model: research mentoring and professional/career mentoring
UCLA	Faculty mentors	Health and policy management; foundations of MCH; summer institute	Requirement to join a service learning organization	No	N/A	Weekly workshops on professionalism and academic guidance; summer internships
USF	Academic mentoring	2 Undergraduate MCH-public health courses; 2 summer institutes	Encouraged and provided with supports; part of portfolio for graduate school application	Development of a hypothetical research project leading to poster	MCH symposium	Research mentoring; 1:1 support for graduate school preparation; Univ health research day; conference attendance
UWM	Discipline mentors; peer mentors; research mentors	Public health 101; client diversity in healthcare; interdisciplinary research in MCH-I and II; MCH 1-social determinants of health; MCH 2-families and leadership; MCH 3-public and population health	Integrated service leadership; title V-related agency partnerships; family mentoring	1 year of mentored research leading to abstract and poster	University undergrad research symposium (annual); national conferences	Discipline (career) mentoring; research mentoring; GRE test prep; 1:1 support for graduate school preparation; resume/employment support; national conference in career field

Expectations of the MCHPTPs that distinguish these training programs from more general pipeline initiatives include the requirements and opportunities for training within the larger MCH network. Table 3 provides a summary of how each MCHPTP responds to MCH resources and opportunities to achieve the comprehensive offerings of MCH Pipeline training.

**Table 3:** Pipeline training specific to MCH

Program	Application/integration of MCH leadership competencies	Link to long-term MCHB training	Description of link to long-term MCHB training	Making Lifelong Connections conference	MCHWDC Title V Summer Internship
ASU	Integrated into summer internship and into leadership courses	UAB MCH leadership network; GA-LEND	Collaborative partnership to provide knowledge and opportunities for entry into the various MCH training programs within the network. Providing experiences and speakers with MCH disciplines/backgrounds	Financial and mentoring support for student involvement	Students accepted
BCM-TSU	Adapted for internship experience; used for course objectives and leadership development	BCM-COE; Healthy Start	Collaborative research opportunities; community outreach; resources	N/A	Students accepted
KKI	Addressed in all aspects of program; MCHPH-CCS pre-/post-test	JHU-LEND	Collaborative training events and advocacy activities	N/A	Students accepted
UCLA	Integrated into course (esp cultural competency and critical thinking)	UC-LEND; child and family health leadership training program	Pipeline students network with all MCH trainees, work together on projects, and participate in MCH Interest Group. Graduate students serve as mentors to Pipeline students	Financial and mentoring support for student involvement	Students accepted
USF	Used for program development and evaluation	USF-COE	If student remains at USF, opportunity to apply to COE	Financial and mentoring support	Students accepted
UWM	Used for program development and evaluation; course objectives; and tracking student leadership development	WI-LEND; WI-PPC; WI-Catalyst; UIC-COE	Collaborative training events across programs to leverage geographic offerings and to create interaction points across programs to support trainee awareness of the continuum of MCH training opportunities; supports recruitment of pipeline students into graduate training programs	Financial and mentoring support for student involvement	Students accepted

*LEND* leadership and education in neurodevelopmental disabilities, *COE* center of excellence, *PPC* pediatric pulmonary center, *MCH-PH-CCS* maternal and child health/public health core competency, *GA* Georgia, *JHU* Johns Hopkins University, *UIC* University of Illinois at Chicago

## Discussion

### Comprehensiveness of the MCH Pipeline Training Program

To achieve the objective of a more diverse and representative workforce, all MCHPTPs are required to provide comprehensive training, including a variety of learning experiences (e.g. didactic, experiential, mentoring, and peer exchange), as well as support for academic success, leadership development, research development, and planning for MCH careers (MCHB/NOFO, 2016). Indeed, the MCHPTP is not simply an enrichment program; it is a comprehensive program that seeks to reduce barriers to completion of an undergraduate degree while also intentionally supporting progression to graduate school or directly into the MCH workforce. Several themes emerged from the survey of programs that reinforce the comprehensive nature of the MCHPTPs and the successful outcomes from these programs, including strategies for effective recruitment, support of student persistence in the academic pipeline, and the intentionality of pipeline-to-workforce training.

### Recruitment

There was no “one-size-fits-all” approach to recruitment of students for these Pipeline programs, nor was recruitment a simple process. Indeed, multiple concurrent approaches seem to be required, regardless of program design or location. Programs reported working with and through campus offices of diversity and inclusion, student organizations targeting URM students, service or community outreach organizations, as well as presenting to students in feeder classes and prioritizing direct recruitment of students by faculty and/or current trainees. Several programs identified that direct recruitment resulted in the best matches of students to programs.

### Supporting Student Persistence in the Academic Pipeline

The MCHPTPs recruit and support students from economically and/or educationally disadvantaged backgrounds, many of whom are also first-generation college students. These students experience especially unique challenges. To meet these challenges, MCHPTPs are expected to address the emotional and social as well as academic and financial needs of students.

#### Financial Support

Financial support is a component of most pipeline programs. While this is helpful for all students, especially students who might otherwise be unable to complete their undergraduate education and/or unable to afford application to graduate school (Camacho et al., 2017), for most students, the financial support is not the driving force to engaging and staying engaged in the MCHPTPs. Students cite experiential learning, mentoring, targeted preparation for graduate school, and being part of a healthcare program for students of color as their reasons for staying engaged (as reported by program directors for insights gained from administering these programs).

#### Mentoring

Mentoring by faculty is a primary reason that students seek pipeline-type programs (Guerrero et al., 2015). Mentoring relationships can be critical to promoting a student’s sense of self-worth, self-efficacy, and confidence. This may be especially critical to support academic persistence among first generation college students (Schultz et al., 2011). Meaningful mentoring, including close academic advising, research mentoring, and mentoring for graduate school and/or progression to workforce and career planning were reported for all MCHPTPs (see Belcher et al﻿., in review). One-on-one relationships with program faculty were reported to include mentoring around professionalism, emotional intelligence, resiliency, and managing challenges related to family of origin attitudes toward education (Kuo et al., 2015). It is not unusual for students who are first generation US citizens to be supporting their families of origin, while also working to put themselves through school (Garner & Holley, 2011). Mentors will play a critical role in supporting these students as they navigate the potentially unrealistic expectation for degree completion in 4 years. Increasingly, mentors also have to be prepared to address issues such as imposter syndrome, stereotype threat, structural racism, student homelessness and food insecurity, as well as trauma backgrounds and mental health challenges (Wyatt et al., 2019). Mentoring can be critical to students accessing and engaging with impactful resources that allow them to persist in the academic pipeline.

#### Belonging and Social Support

Research has shown that academic and financial support alone are not sufficient; URM students also need to experience being part of a community (Allen-Ramdial & Campbell, 2014; Summers & Hrabowski, 2006). Belonging and social identify are critical motivators of achievement (Cohen & Garcia, 2008), and the end result of group identification may be a sense of inclusion of authentic self within the healthcare fields (Ahern-Dodson et al, 2020). Four out of five of the MCHPTPs use a cohort or group model for training, within which students have the opportunity to develop relationships with peers who have similar interests and motivations, and who, in many cases, may have had similar life experiences. Additionally, all of the MCHPTPs have predictable and frequent touch points in the forms of meetings, dinners, seminars, and community activities to concurrently address student self-growth and their knowledge of MCH, while also reinforcing their connections to peers and mentor support.

#### Research

Research exposure for undergraduate students has been found to influence the growth trajectories of students’ intentions to pursue science and healthcare fields and to persist with their academic program (Schultz et al, 2011). All of the MCHPTPs expose students to MCH research, and several engage students in the research process and products including poster presentations at campus, regional and national research conferences (see Olaleye et al.,﻿ in review). Mentored research experiences add another opportunity for students to develop relationships with faculty who will support their persistence in the academic pipeline (Alegria et al., 2019).

### Pipeline to Graduate Education and MCH Workforce

The MCHPTPs have 2 related primary objectives: supporting successful progression of undergraduate students from URMs into graduate training in the MCH fields and supporting MCH workforce development (MCHB/NOFO, 2016). The pipeline-to-workforce can be direct or indirect (via graduate school), where support for graduate school readiness is of paramount importance. To support the challenge of this academic step, the MCHPTPs offer concrete professional development opportunities and even assistance with preparation of graduate school materials. For some students, however, the challenge is more than just the fear of being competitive for admission to graduate school or knowing how to navigate this stage of education; first-generation undergraduate students often encounter cultural challenges related to the impact of educational mobility on the role assignments and dynamics of their families (Gardner & Holley, 2011). Even if graduate education is required for the field they have worked toward, the decision to pursue additional education for their career may augment their struggle with living with disparate world views from their families of origin and can come at a tremendous cost to the student who chooses education over family expectations (Gardner & Holley, 2011). The role of MCHPTPs in addressing the individual emotional and social needs of students becomes particularly relevant at this pipeline-to-graduate-school juncture, as students navigate the impact of what more education may mean for their immediate place in their families and communities. The need for wrap-around support to achieve the actual pipeline-to-graduate-school transition is critical for many first-generation students persisting in the pipeline (Kuo et al., 2015).

#### Exposure to Other MCH Training and Development

The requirement of MCHPTPs to have at least one meaningful linkage to a long-term MCH graduate training program enhances the undergraduate training experience and ideally facilitates future graduate placement into MCH programs (MCHB/NOFO, 2016). All of the MCHPTPs collaborate with long-term MCH graduate training programs at their institutions, and several have additional linkages to long-term training at partner or other regional institutions. Exposure to long-term MCH training as well as participation in other supportive programs, including the Title V Summer Internship Program and the Making Lifelong Connections conference, create the overlap of social and professional networks understood to be critical to the persistence of students in pursuit of professional education (Ahern-Dodson et al, 2020).

## Conclusions

The urgency to achieve a diverse and representative MCH workforce is magnified by the rapidly-changing demographics of the U.S population. The MCHPTPs are a response to the critical need to diversify the MCH workforce and reduce health disparities in MCH populations, by increasing the capacities of its trainees to enter the MCH workforce. This paper characterized the models and components of training across the 6 funded undergraduate MCHPTPs, to understand the strategies that underlie pipeline training to impact educational attainment and workforce outcomes. The outcomes of these MCHPTPs is measured by number of students who move from undergraduate to graduate education and ultimately into the MCH workforce (see Fernandes et al﻿., 2022). These programs, however, do more than just function as a conduit to graduate education and the MCH workforce; they “fulfill the promise of the pipeline” (Peterson, 2019), by preparing the next generation of MCH leaders.

## Data Availability

Not applicable.
